# Supplementary material for: Different Nitrogen Consumption Patterns in Low Temperature Fermentations in the Wine Yeast Saccharomyces cerevisiae
Source: Foods. 2024 Aug 13;13(16):2522. doi: 10.3390/foods13162522 (PMC11354071; doi:10.3390/foods13162522)
Supplement: Supplementary file 1 [file foods-13-02522-s001.zip › foods-3135365-supplementary.pdf]

foods

28ºC

P5	P24	P5	P24	P5	P24
60 mg/L N	60 mg/L N	140 mg/L N	140 mg/L N	300 mg/L N	300 mg/L N
Alanine	Alanine	Aspartate	Lysine	Lysine	Lysine
Glutamate	Aspartate	Glutamate	Methionine	Methionine	Methionine
Glutamine	Glutamate	Histidine	Leucine	Leucine	Leucine
Glycine	Glutamine	Isoleucine	Phenylalanine	Tryptophan	Tryptophan
Histidine	Glycine	Leucine	Isoleucine	Aspartate	Phenylalanine
Isoleucine	Histidine	Lysine	Histidine	Histidine	Proline
Leucine	Isoleucine	NH4+	Aspartate	Phenylalanine	Cysteine
Lysine	Leucine	Phenylalanine	Tryptophan	Threonine	Arginine
NH4+	Lysine	Methionine	Proline	Proline	Histidine
Phenylalanine	Methionine	Threonine	NH4+	Arginine	Aspartate
Threonine	Phenylalanine	Arginine	Glutamate	Cysteine	Glutamate
Tyrosine	Serine	Serine	Threonine	Serine	Isoleucine
Valine	Threonine	Tryptophan	Serine	Glutamine	Glutamine
Tryptophan	Tryptophan	Glutamine	Cysteine	Isoleucine	Threonine
Methionine	Tyrosine	Valine	Glutamine	Glutamate	Serine
Arginine	Valine	Proline	Valine	NH4+	Tyrosine
Serine	NH4+	Cysteine	Arginine	Tyrosine	NH4+
Proline	Arginine	Tyrosine	Tyrosine	Glycine	Valine
Aspartate	Proline	Alanine	Glycine	Valine	Alanine
Cysteine	Cysteine	Glycine	Alanine	Alanine	Glycine

15ºC

P5	P24	P5	P24	P5	P24
60 mg/L N	60 mg/L N	140 mg/L N	140 mg/L N	300 mg/L N	300 mg/L N
Alanine	Alanine	Histidine	Histidine	Lysine	Isoleucine
Arginine	Glutamate	Leucine	Isoleucine	Methionine	Leucine
Aspartate	Glutamine	Lysine	Leucine	Leucine	Lysine
Glutamate	Glycine	Methionine	Lysine	Histidine	Methionine
Glutamine	Histidine	NH4+	Methionine	Tryptophan	Phenylalanine
Glycine	Isoleucine	Isoleucine	Phenylalanine	Threonine	Threonine
Histidine	Leucine	Threonine	NH4+	Serine	Histidine
Isoleucine	Lysine	Serine	Arginine	Arginine	Serine
Leucine	Methionine	Aspartate	Threonine	Phenylalanine	Tryptophan
Lysine	NH4+	Arginine	Serine	Isoleucine	Arginine
Methionine	Phenylalanine	Phenylalanine	Tryptophan	Cysteine	Valine
NH4+	Serine	Tryptophan	Aspartate	Proline	Aspartate
Phenylalanine	Threonine	Glutamine	Proline	Glutamine	Cysteine
Serine	Tryptophan	Glutamate	Glutamate	Aspartate	Glutamine
Threonine	Tyrosine	Valine	Valine	NH4+	Proline
Tryptophan	Valine	Proline	Glutamine	Tyrosine	NH4+
Tyrosine	Arginine	Cysteine	Cysteine	Valine	Tyrosine
Valine	Proline	Alanine	Tyrosine	Glutamate	Glutamate
Proline	Aspartate	Tyrosine	Alanine	Alanine	Alanine
Cysteine	Cysteine	Glycine	Glycine	Glycine	Glycine

Figure S1. Ranking of amino acid consumption by strains P5 and P24 at 28 and 15°C. Consumption percentages. Yellow (75-100%), Green (50-75%); Blue (25-50%) and Pink (0-25%). .
